# Supplementary material for: Self‐Adjusting Engineered Probiotic for Targeted Tumor Colonization and Local Therapeutics Delivery
Source: Adv Sci (Weinh). 2025 May 28;12(31):e06486. doi: 10.1002/advs.202406486 (PMC12376540; doi:10.1002/advs.202406486)
Supplement: Supplementary file 1 — Supporting Information [file ADVS-12-e06486-s001.docx]

**Supporting Information**

**Self-adjusting Engineered Probiotic for Targeted Tumor Colonization and Local Therapeutics Delivery**

Zhen-Ping Zou,^1^ Xin-Ge Wang, ^1^ Xuan-Ren Shi, ^1^ Shu-Ting Sun, ^1^ Jing Mi, ^1^ Xiao-Peng Zhang, ^1^ Bin-Cheng Yin, ^1^ Ying Zhou, ^1^* Bang-Ce Ye ^1,2^*

^1^Laboratory of Biosystems and Microanalysis, State Key Laboratory of Bioreactor Engineering, East China University of Science and Technology, Shanghai 200237, China

^2^Institute of Engineering Biology and Health, Collaborative Innovation Center of Yangtze River Delta Region Green Pharmaceuticals, College of Pharmaceutical Sciences, Zhejiang University of Technology, Hangzhou 310014, Zhejiang, China

*Correspondence: zhouying@ecust.edu.cn (Y.Z.), bcye@ecust.edu.cn (B.-C.Y.)


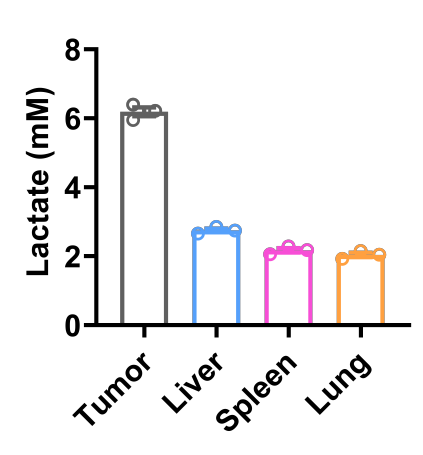


**Figure S1.** **Lactate levels in MC38 tumors and healthy organs.** MC38 cells were injected at a volume of 100 μL (5×10^5^ cells). Tumors were grown to an average of approximately 80-100 mm^3^ and then collected tumors and organs. Lactate concentrations were detected by lactate colorimetric assay kits (mean ± SEM, n=3).


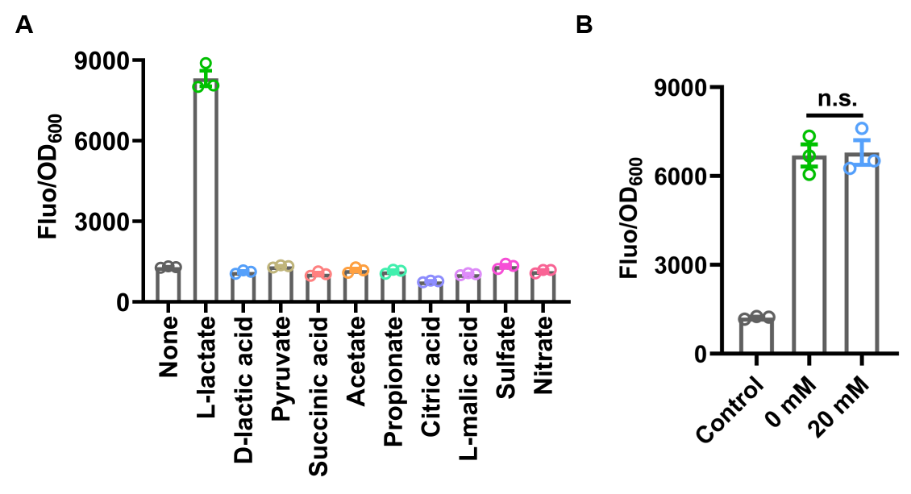


**Figure S2. Specificity test and glucose inhibition effect analysis of the L-lactate responsive biosensor, L1032.** (A) The specificities of L1032 were determined, and all ligands were tested at a concentration of 10 mM (mean ± SEM, n=3). (B) L1032 was cultured in presence of 10 mM L-lactate and 0 or 20 mM glucose. No decrease in response indicates no inhibition by the added glucose (mean ± SEM, n=3).


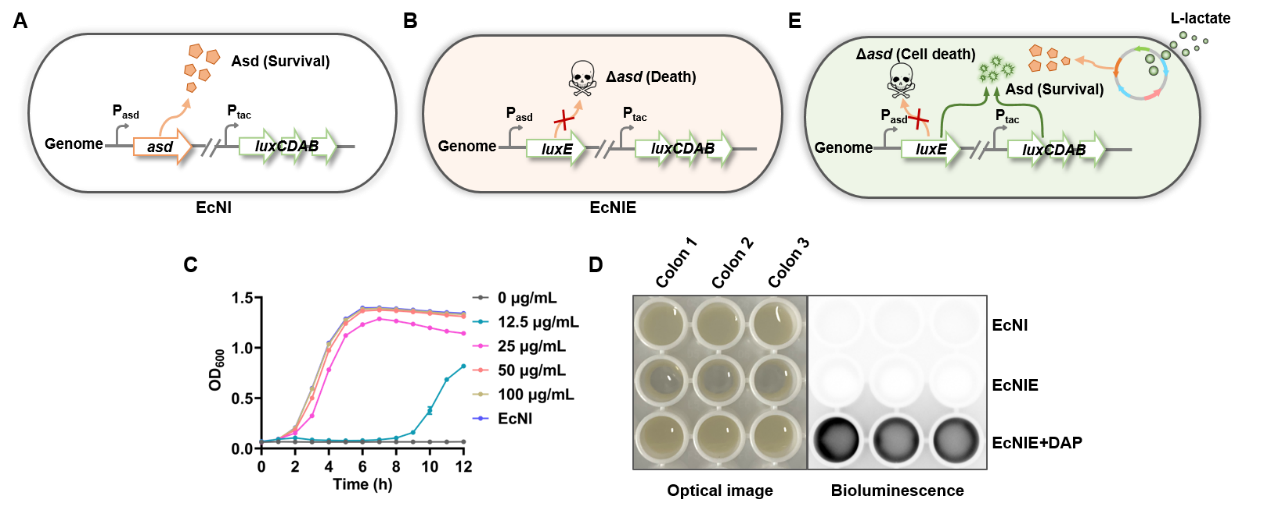


**Figure S3. Construction and characterization of a EcN strain with the *asd* gene knockout.** (A) Schematic diagram of EcNl strain. (B) Schematic diagram of EcNlE strain, which the *asd* gene has been replaced by *luxE*. (C) Growth ability of EcNlE strain (mean ± SEM, n=3). (D) Visualization of EcNlE strain growth through optical image and bioluminescence. (E) Schematic diagram of a engineered EcN strain, which growth is regulated by L-lactate.


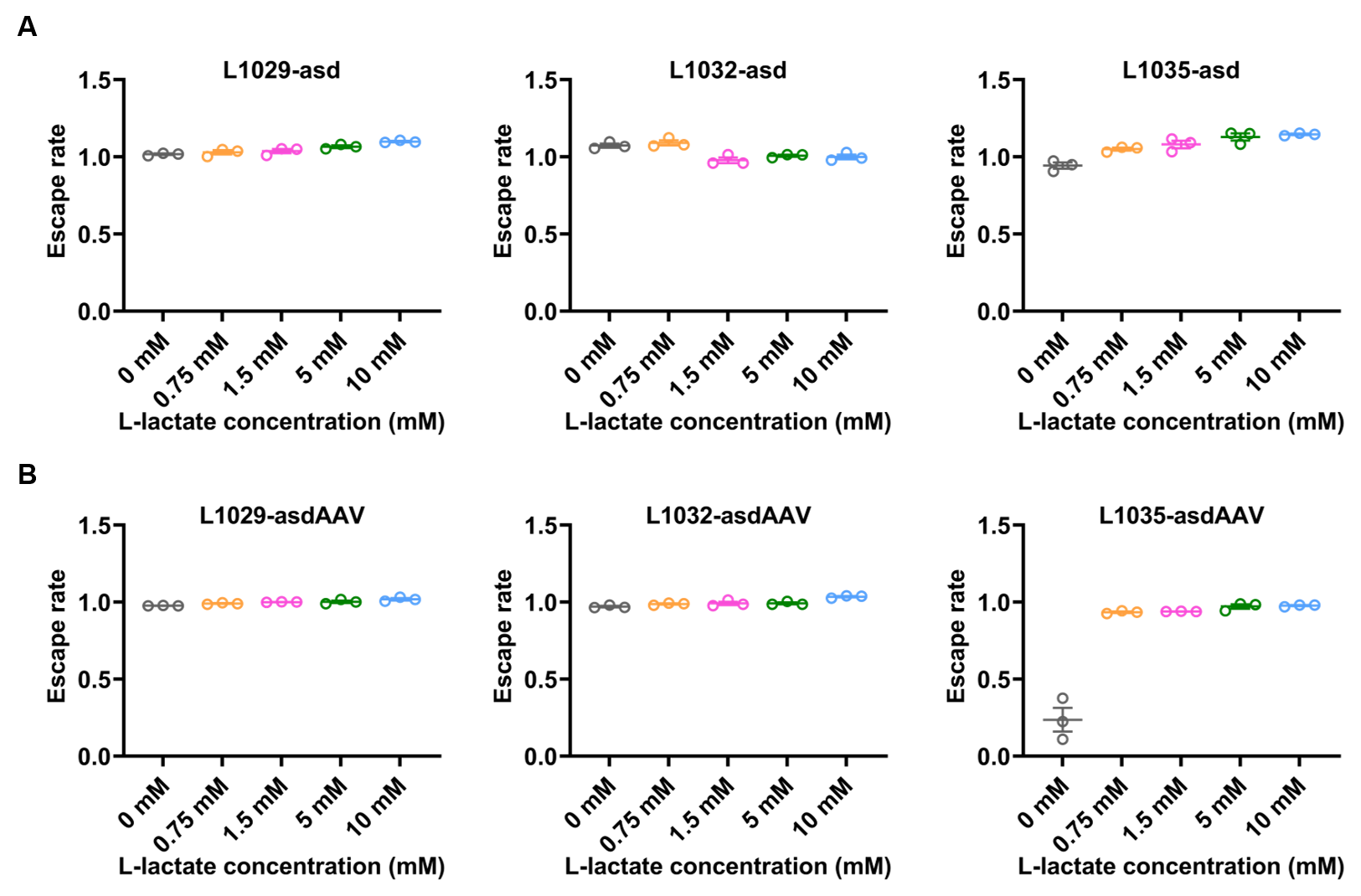


**Figure S4.** **Coupling a L-lactate biosensor with bacterial growth via the expression of an essential gene *asd*.** Characterization of some biocontainment variants on the escapee rate (mean ± SEM, n=3).


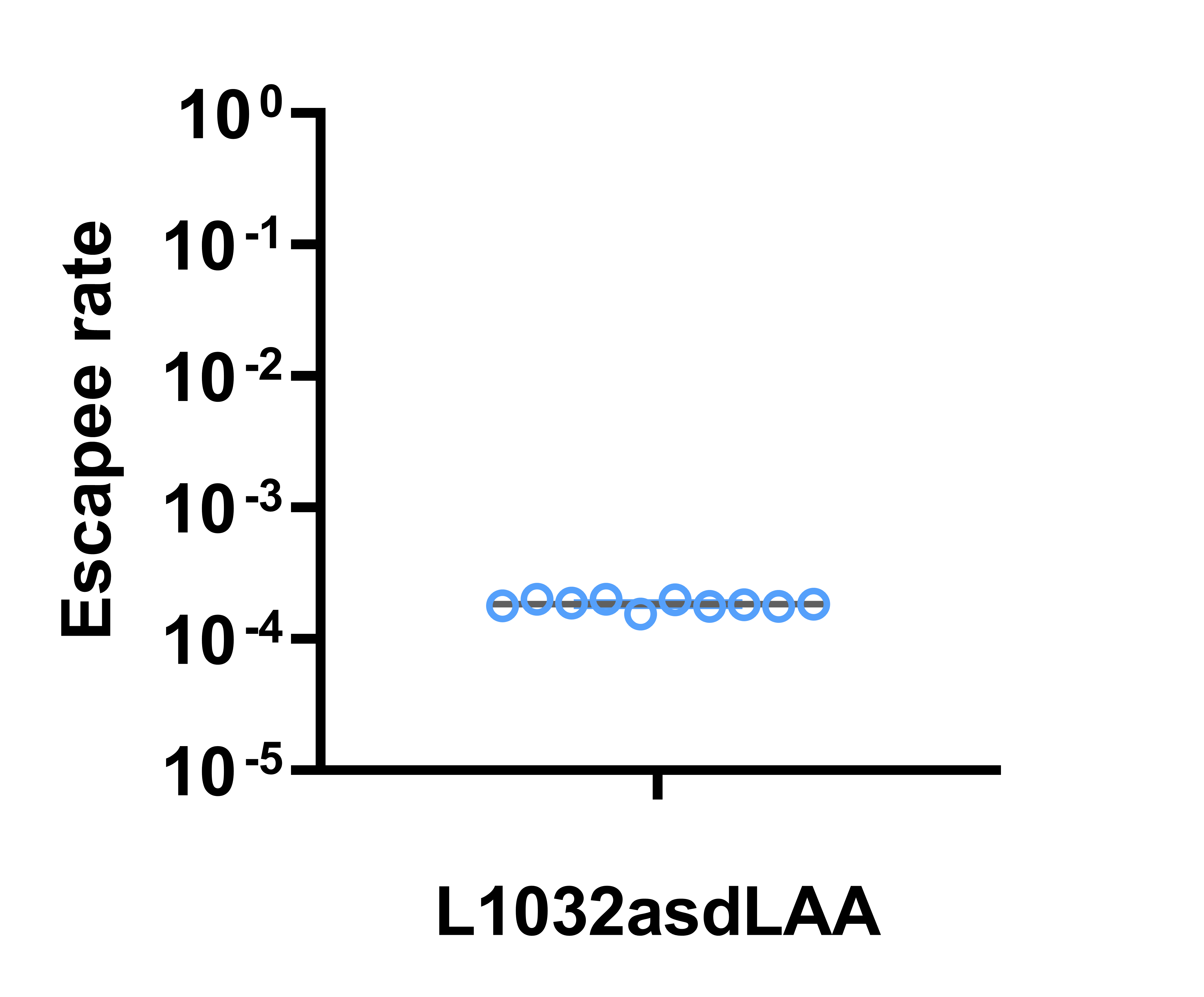


**Figure S5. Characterization of L1032asdLAA on the basis of the “escapee rate”.** “Escapee rate” defined as the ratio between colonies grown in non-permissive (0 mM lactate) and permissive (10 mM lactate) conditions. The cultures were inoculated with the same initial density into 5 mL of medium either without lactate or with 10 mM lactate and incubated for 8 h, and plated on LB agar plates with added 100 μg/mL DAP, after which colonies were counted the next day (mean ± SEM, n = 10).


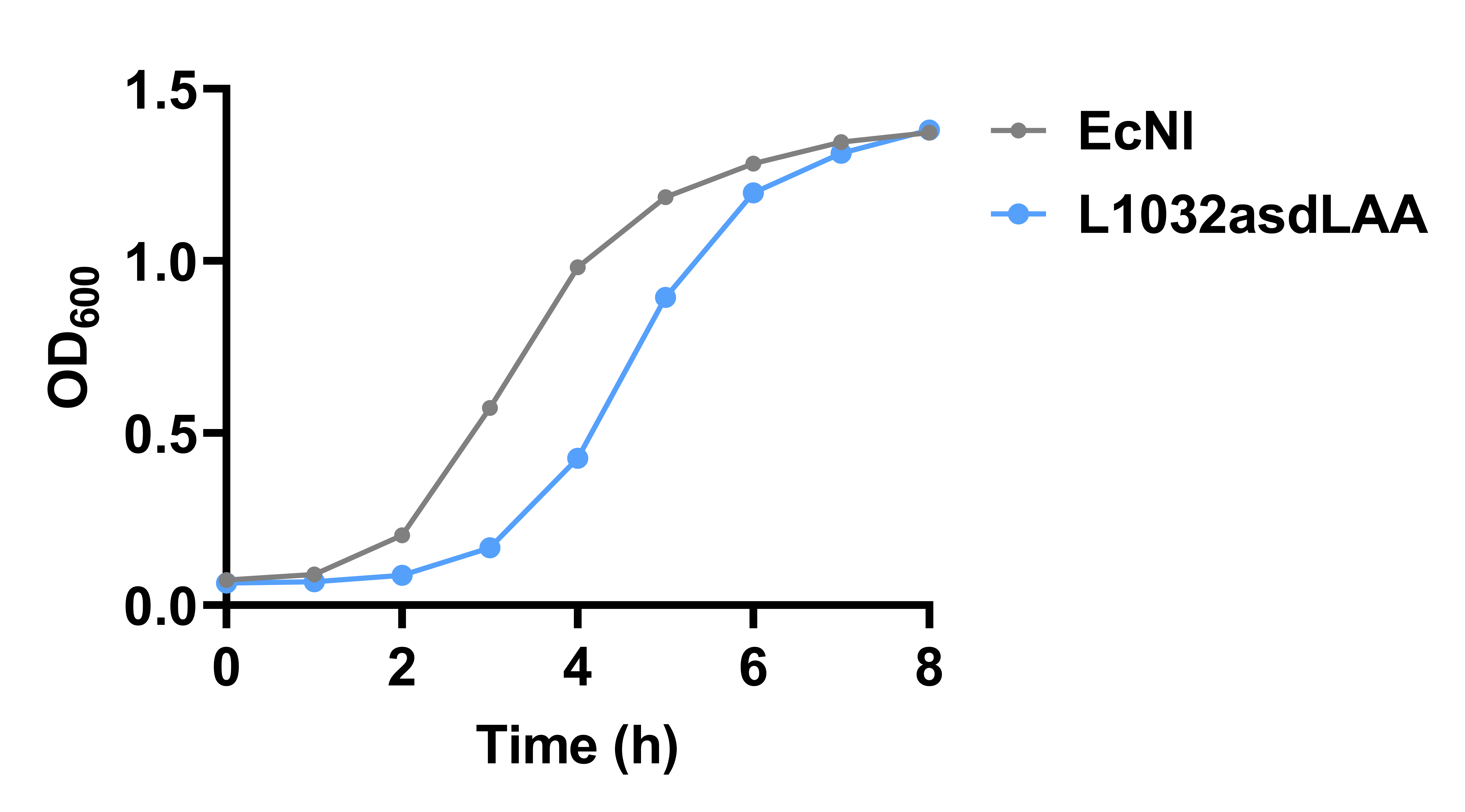


**Figure S6. The growth curves of EcNl and L1032asdLAA strains.** EcNl strain grows in a medium containing 50 μg/mL, while L1032asdLAA grows in a medium containing 10 mM lactate (mean ± SEM, n = 3).


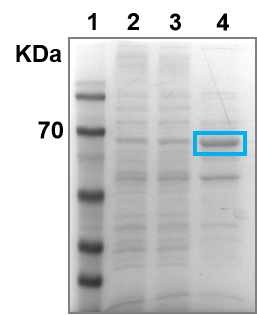


**Figure S7. Expression and purification of truncated Coa coagulase.** Lane 1 is the Marker, Lane 2 is the total protein, Lane 3 is the soluble protein, and Lane 4 is the purified protein.

**
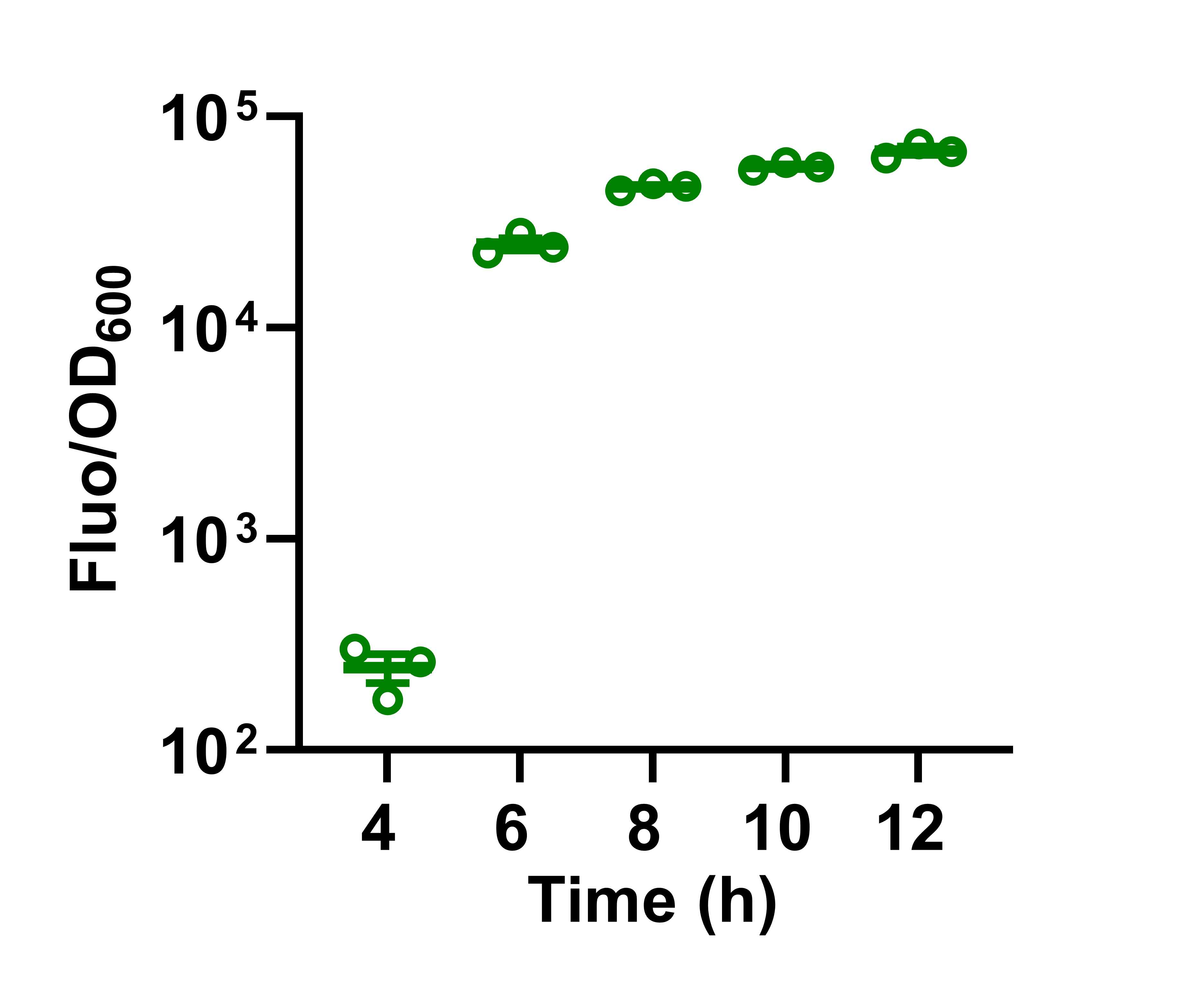
**

**Figure S8.** **Characterization of quorum sensing elements.** The fluorescence signal of EcNl strain containing a quorum-sensing sensor increases with the increasing bacterial concentration (mean ± SEM, n=3).


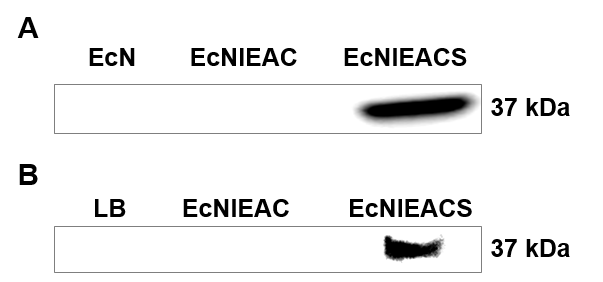


**Figure S9. Detection of the expression of SAH by WB.** (A) Analysis of SAH in bacterial lysates. The bacterial culture was used for WB analysis after 12 h of culture. (B) Analysis of SAH in bacterial culture supernatants. EcNlEAC and EcNlEACS culture supernatants were collected by centrifugation for WB analysis after 12 h of culture.


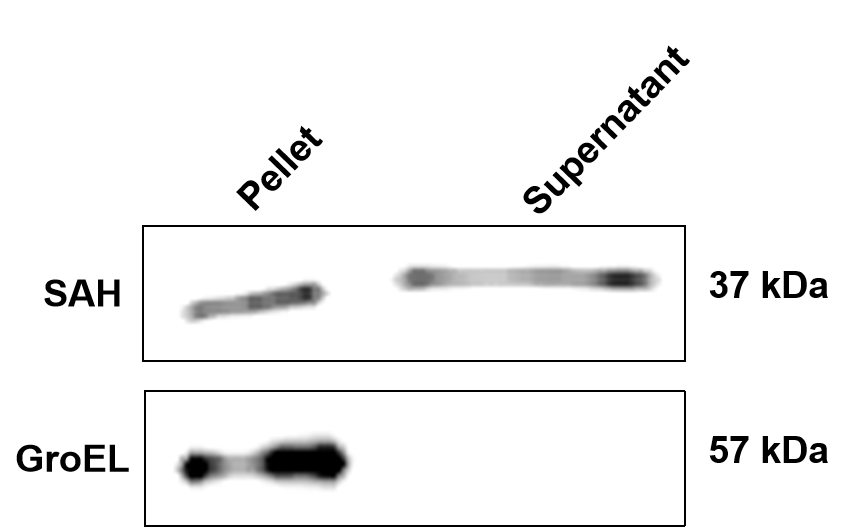


**Figure S10. Secretion assays of EcNlEACS to secrete SAH.** Immunoblots labeled with anti-SAH (Abcam: ab190467) or anti-GroEL (Abcam: ab318970) antibodies are shown. The highly abundant cytosolic protein (GroEL) of bacterial cell was not detected in the supernatant fractions. The culture supernatant was concentrated 5-fold before WB detection.

**
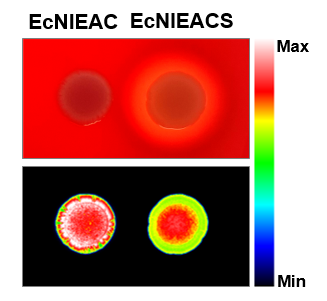
**

**Figure S11.** **Visualization of EcNlEACS strain lysing red blood cells.** Add 10 μL EcNlAC (10^9^ CFU) and EcNlACS (10^9^ CFU) cultures to the surface of the blood agar plate, continue to culture at 37℃ for 12 h, and then image. Clear zone around the EcNlACS colonies indicating the expression of SAH.

**
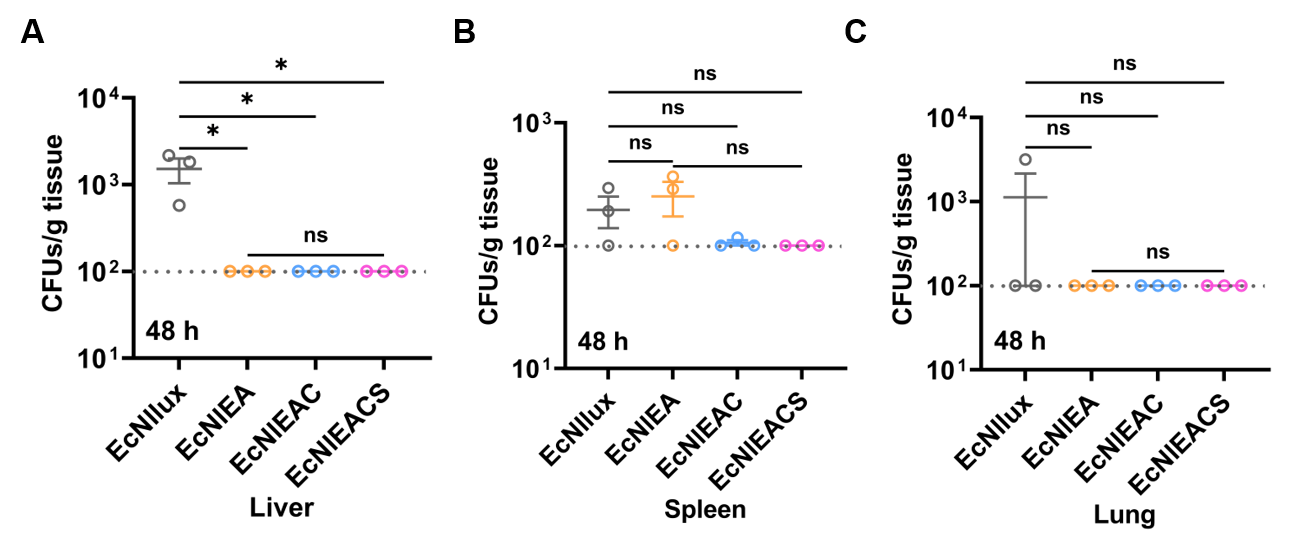
**

**Figure S12.** **Selective colonization of engineered EcN.** Quantification of bacterial colonization in different organs (liver, spleen, lung) harvested from healthy mice after inject bacterial for 48 h. LOD=1×10^2^ CFU/g (mean ± SEM, n=3).


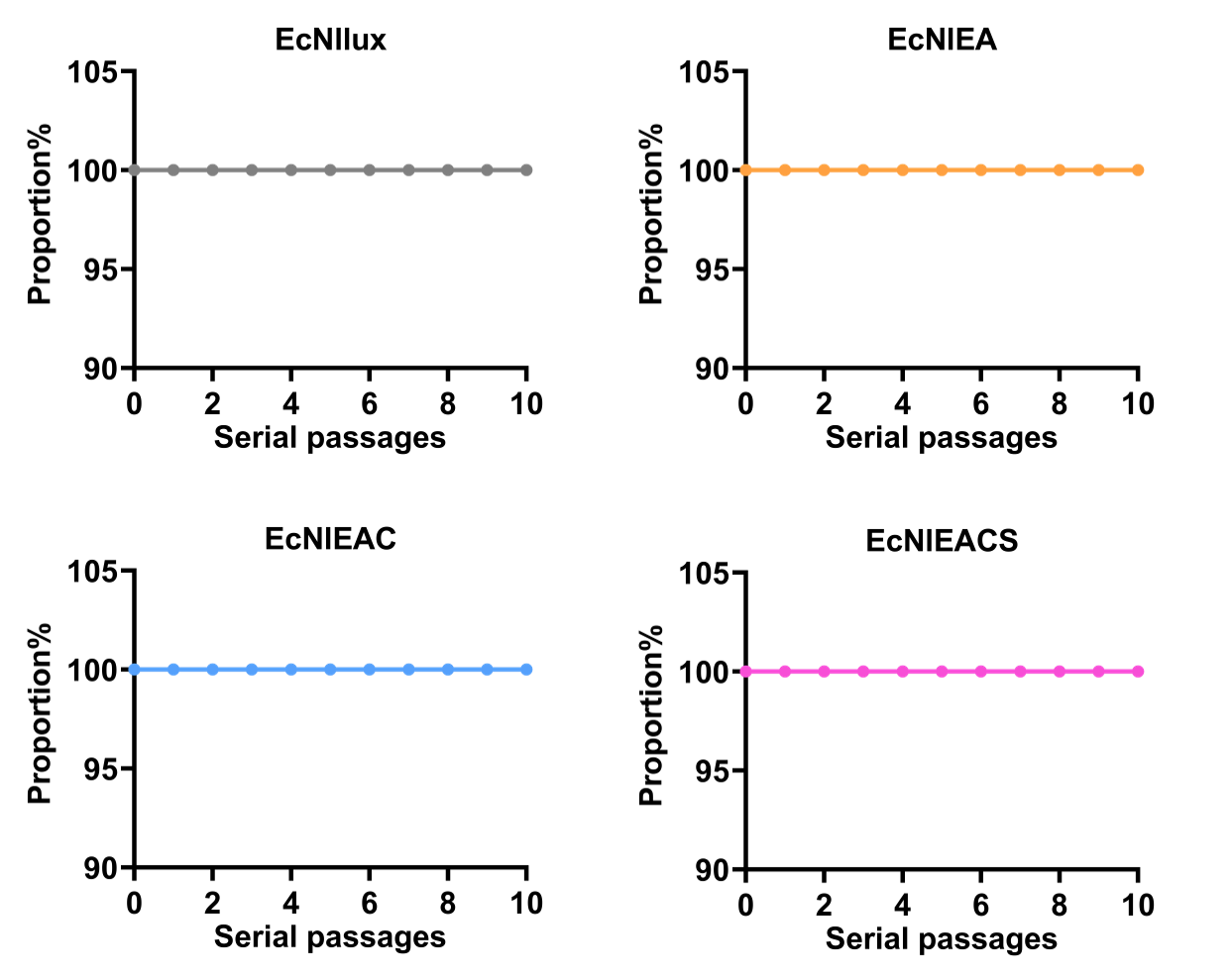


**Figure S13. Plasmid stability analysis.** Strains were passaged (12 h per passage) without resistance stress for 10 passages, and plasmid loss rates were subsequently analyzed through resistance screening (n=3).


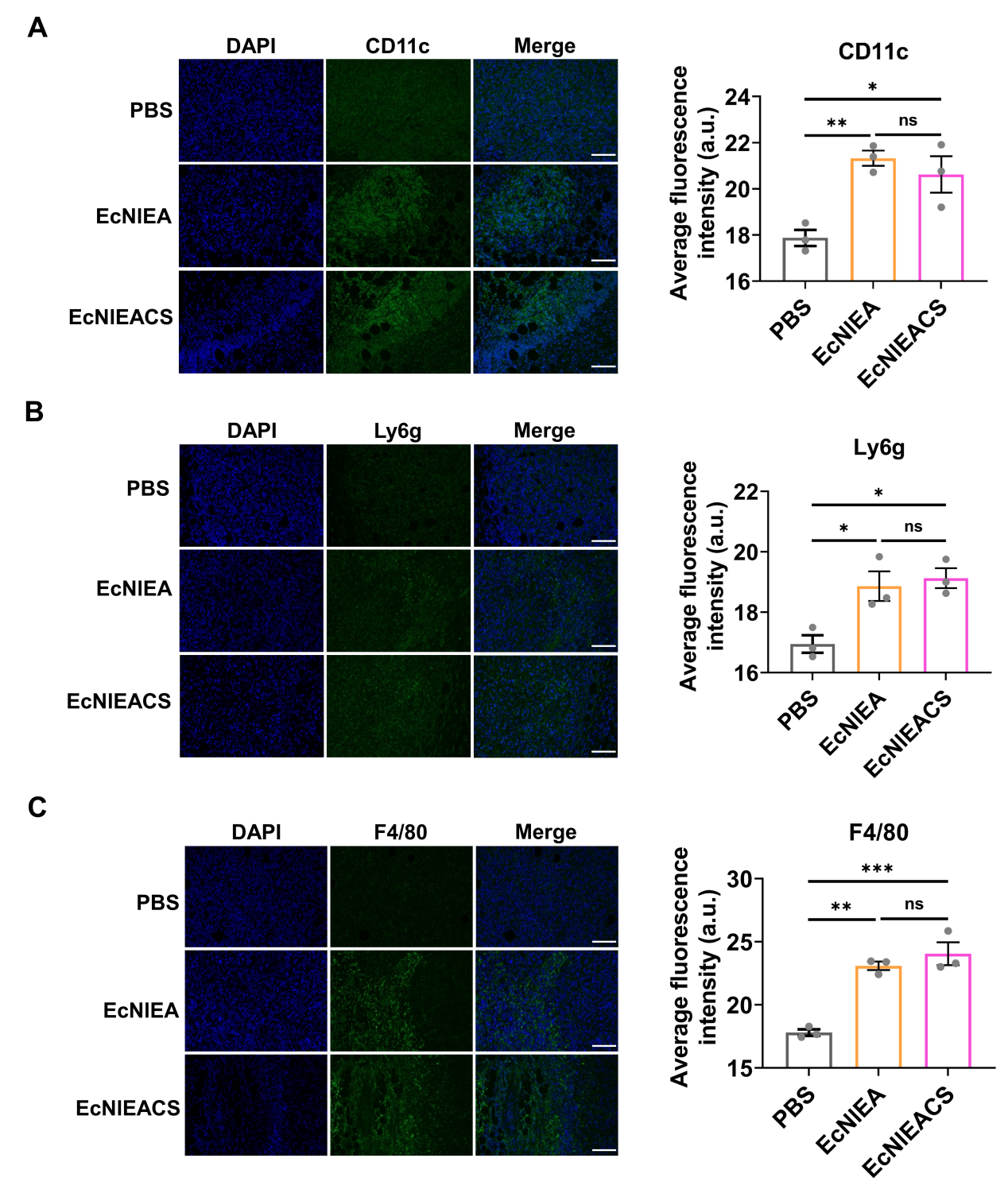


**Figure S14. Analysis of immune cells using immunofluorescence staining.** (A) CD11c: Dendritic cell marker. (B) Ly6g: Neutrophil marker. (C) F4/80: Macrophage marker. All tumors samples were collected from MC38-bearing mice at 24 h after PBS or bacterial infection (5.0 × 10^7^ CFU). Average fluorescence intensity was quantified by ImageJ. Statistical significance was determined by a one-way ANOVA (mean ± SEM, n=3), *p ≤ 0.05, **p ≤ 0.01, ***p ≤ 0.001. Scale bars = 100 μm.


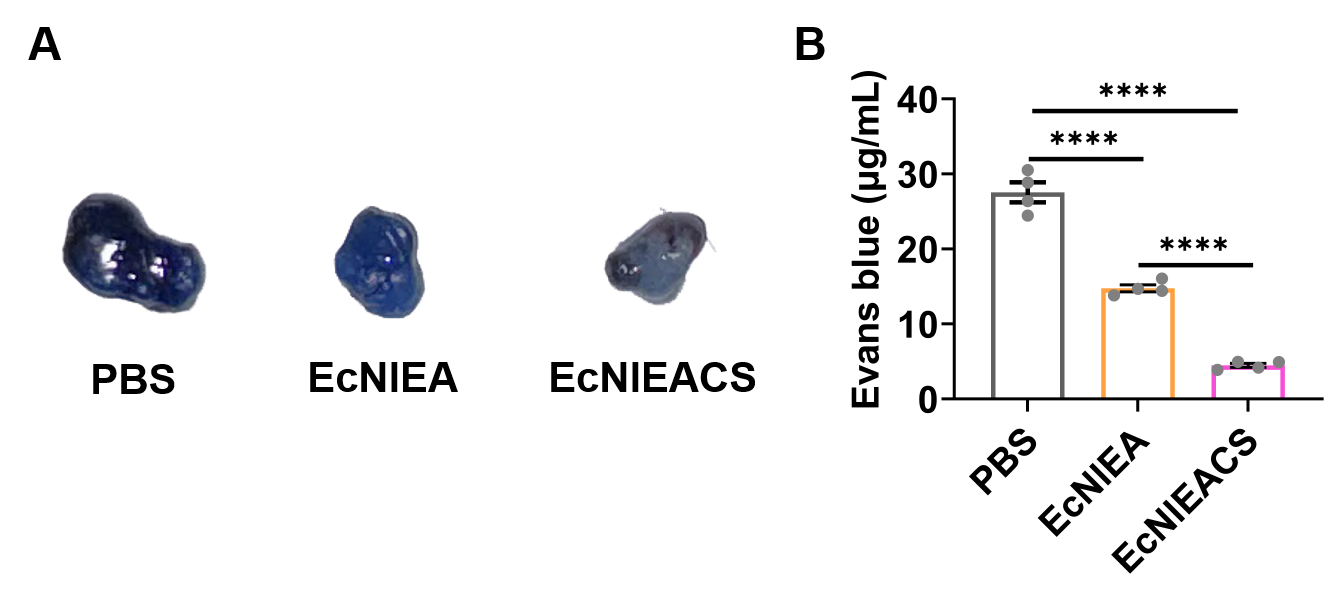


**Figure S15. Evans blue content and corresponding tumor tissue images in the different treatment groups.** 5% Evans blue was administered intravenously 24 h after the different treatments. Three hours later, the mice were sacrificed, and tumor tissues were harvested for imaging. The tumors were then incubated in formamide for 24 h to extract the Evans blue, and absorbance at 620 nm was measured. The concentration of Evans blue in the tumors was quantified using a pre-established standard curve. Statistical significance was determined by a one-way ANOVA, ****p ≤ 0.0001, mean ± SEM, n = 4.


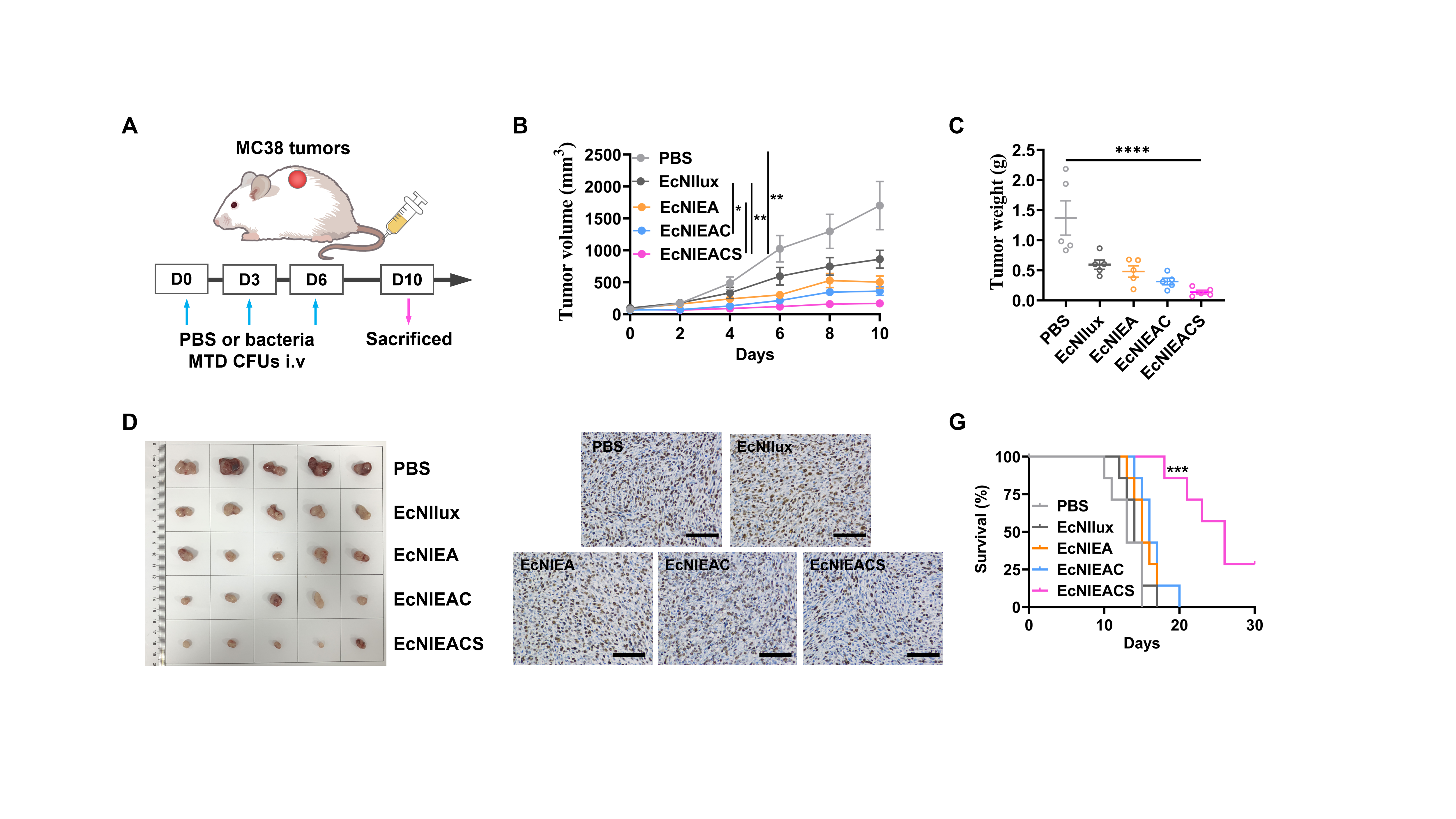


**Figure S16.** **Immunohistochemistry analysis of MC38 tumor sections stained with Ki67 in PBS controls and groups treated with engineered EcN**. The sections were obtained from tumors showed in Figure 8D. Scale bars = 100 μm.

**
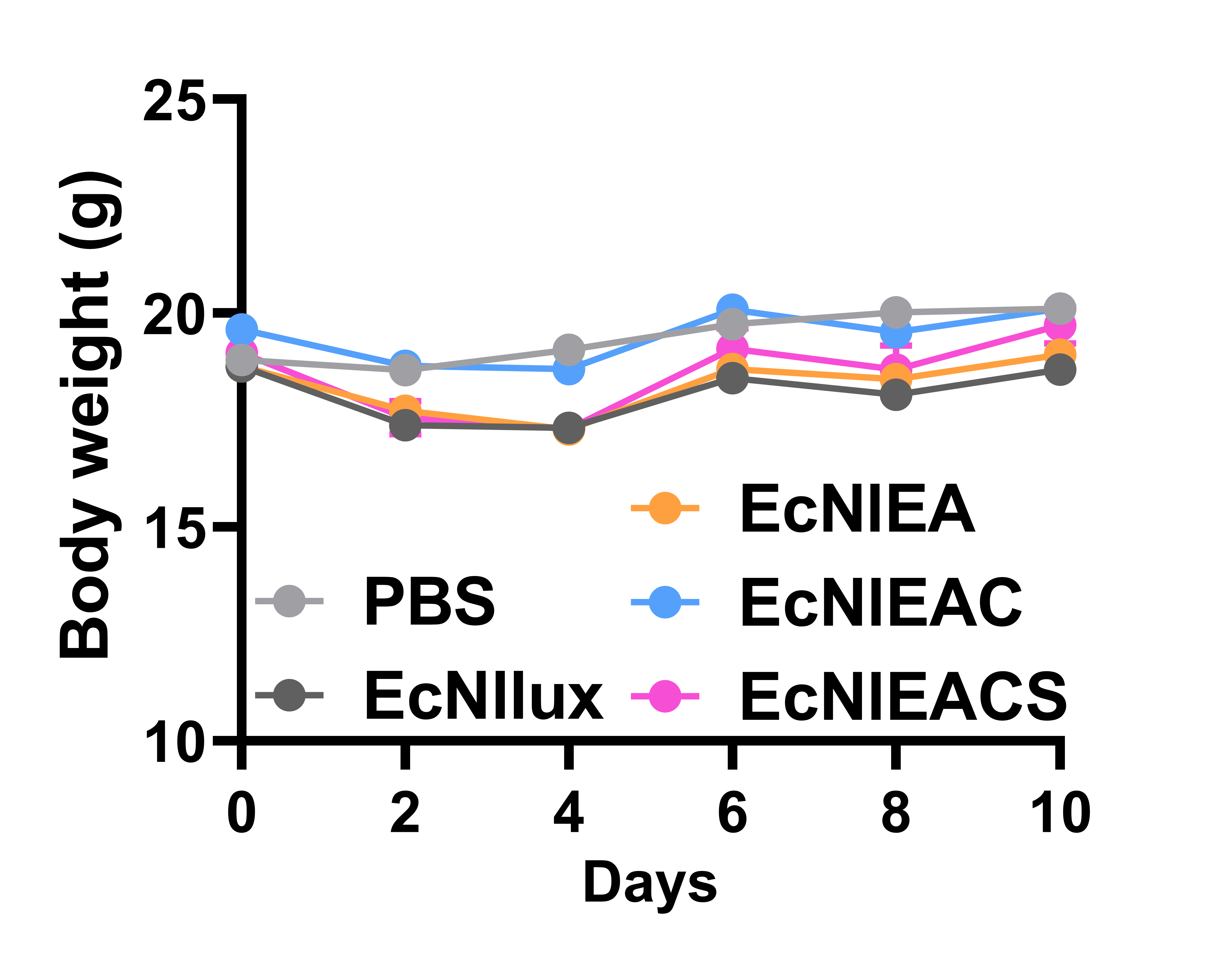
**

**Figure S17.** **Mouse weight change during treatment.** During the treatment process, the weight of the mice was measured every two days.

**Table S1** Summary of the strains used in this study.

| Lable | Characteristics | promoter and RBS of *lldR* | Promoter and RBS of *sfGFP* | promoter of *luxAB* | promoter of *luxCD* | promoter of *luxE* | promoter and RBS of *asd* | promoter and RBS of *coa* | promoter and RBS of *luxR* | RBS of *luxI* | Promoter of *SAH* |
| --- | --- | --- | --- | --- | --- | --- | --- | --- | --- | --- | --- |
| AL | *araC, lldR, sfGFP* | P_ara_- RBS_ara_ | P_tacO_-B0035 | N/A | N/A | N/A | N/A | N/A | N/A | N/A | N/A |
| ALO | *araC, lldR, sfGFP* | P_ara_- RBS_ara_ | P_Otac_-B0035 | N/A | N/A | N/A | N/A | N/A | N/A | N/A | N/A |
| ALOO | *araC, lldR, sfGFP* | P_ara_- RBS_ara_ | P_OtacO_-B0035 | N/A | N/A | N/A | N/A | N/A | N/A | N/A | N/A |
| L0435 | *lldR, sfGFP* | P_j23104_- B0035 | P_OtacO_-B0035 | N/A | N/A | N/A | N/A | N/A | N/A | N/A | N/A |
| L0835 | *lldR, sfGFP* | P_j23108_-B0035 | P_OtacO_-B0035 | N/A | N/A | N/A | N/A | N/A | N/A | N/A | N/A |
| L1035 | *lldR, sfGFP* | P_j23110_-B0035 | P_OtacO_-B0035 | N/A | N/A | N/A | N/A | N/A | N/A | N/A | N/A |
| L1029 | *lldR, sfGFP* | P_j23110_-B0029 | P_OtacO_-B0035 | N/A | N/A | N/A | N/A | N/A | N/A | N/A | N/A |
| L1032 | *lldR, sfGFP* | P_j23110_-B0032 | P_OtacO_-B0035 | N/A | N/A | N/A | N/A | N/A | N/A | N/A | N/A |
| L0432 | *lldR, sfGFP* | P_j23104_-B0032 | P_OtacO_-B0035 | N/A | N/A | N/A | N/A | N/A | N/A | N/A | N/A |
| EcNl | EcN::*luxABCD* | N/A | N/A | P_tac_ | P_tac_ | P_j23108_ | N/A | N/A | N/A | N/A | N/A |
| EcNlE | EcN::*luxABCDEΔasd* | N/A | N/A | P_tac_ | P_tac_ | P_j23108_ | N/A | N/A | N/A | N/A | N/A |
| L1029asd | Use EcNlE as the chassis*, lldR, asd* | P_j23110_-B0029 | N/A | P_tac_ | P_tac_ | P_j23108_ | P_OtacO_-B0035 | N/A | N/A | N/A | N/A |
| L1032asd | Use EcNlE as the chassis*, lldR, asd* | P_j23110_-B0032 | N/A | P_tac_ | P_tac_ | P_j23108_ | P_OtacO_-B0035 | N/A | N/A | N/A | N/A |
| L1035asd | Use EcNlE as the chassis*, lldR, asd* | P_j23110_-B0035 | N/A | P_tac_ | P_tac_ | P_j23108_ | P_OtacO_-B0035 | N/A | N/A | N/A | N/A |
| L1029asdAAV | Use EcNlE as the chassis*, lldR, asd-laa* | P_j23110_-B0029 | N/A | P_tac_ | P_tac_ | P_j23108_ | P_OtacO_-B0035 | N/A | N/A | N/A | N/A |
| L1032asdAAV | Use EcNlE as the chassis*, lldR, asd-laa* | P_j23110_-B0032 | N/A | P_tac_ | P_tac_ | P_j23108_ | P_OtacO_-B0035 | N/A | N/A | N/A | N/A |
| L1035asdAAV | Use EcNlE as the chassis*, lldR, asd-laa* | P_j23110_-B0035 | N/A | P_tac_ | P_tac_ | P_j23108_ | P_OtacO_-B0035 | N/A | N/A | N/A | N/A |
| L1029asdLAA | Use EcNlE as the chassis*, lldR, asd-laa* | P_j23110_-B0029 | N/A | P_tac_ | P_tac_ | P_j23108_ | P_OtacO_-B0035 | N/A | N/A | N/A | N/A |
| L1032asdLAA (EcNlEA) | Use EcNlE as the chassis*, lldR, asd-laa* | P_j23110_-B0032 | N/A | P_tac_ | P_tac_ | P_j23108_ | P_OtacO_-B0035 | N/A | N/A | N/A | N/A |
| L1035asdLAA | Use EcNlE as the chassis*, lldR, asd-laa* | P_j23110_-B0035 | N/A | P_tac_ | P_tac_ | P_j23108_ | P_OtacO_-B0035 | N/A | N/A | N/A | N/A |
| L1035coa | Use EcNlE as the chassis, *lldR,* *coa* | P_j23110_-B0035 | N/A | P_tac_ | P_tac_ | P_j23108_ | N/A | P_OtacO_-B0035 | N/A | N/A | N/A |
| L1032asdcoa | Use EcNlE as the chassis, *lldR, asd-laa*, *coa* | P_j23110_-B0032 | N/A | P_tac_ | P_tac_ | P_j23108_ | P_OtacO_-B0035 | P_OtacO_-B0035 | N/A | N/A | N/A |
| EcNlEAC | Use EcNlE as the chassis, *lldR, asd-laa*, *coa* | P_j23106_-B0035 | N/A | P_tac_ | P_tac_ | P_j23108_ | P_OtacO_-RBS36 | P_OtacO_-B0029 | N/A | N/A | N/A |
| EcNlEACS | Use EcNlE as the chassis, *lldR, asd-laa*, *coa, luxI, luxR, SAH* | P_j23106_-B0035 | N/A | P_tac_ | P_tac_ | P_j23108_ | P_OtacO_-RBS36 | P_OtacO_-B0029 | P_j23108_-B0035 | RBS_luxI_ | P_lux_ |

**Table S2** Sequences

| Name | Sequences |
| --- | --- |
| P_ara_ | aaagccatgacaaaaacgcgtaacaaaagtgtctataatcacggcagaaaagtccacattgattatttgcacggcgtcacactttgctatgccatagcatttttatccataagattagcggatcctacctgacgctttttatcgcaactctctactgtttctccat |
| P_j23104_ | ttgacagctagctcagtcctaggtattgtgctagc |
| P_j23108_ | ctgacagctagctcagtcctaggtataatgctagc |
| P_j23110_ | tttacggctagctcagtcctaggtacaatgctagc |
| P_j23106_ | tttacggctagctcagtcctaggtatagtgctagc |
| P_tac_ | ttgacaattaatcatcggctcgtataatg |
| P_tacO_ | ttgacaattaatcatcggctcgtataatgcttgtggtctgaccaatga |
| P_Otac_ | ttgacaatttggtctgaccacgtataatg |
| P_OtacO_ | ttgacaatttggtctgaccacgtataatgcttgtggtctgaccaatga |
| P_lux_ | agcacctgtaggatcgtacaggtttacgcaagaaaatggtttgttatagtcgaatgaattcattaaagaggagaaaggtacc |
| RBS_ara_ | acccgtttttttgggaattcgagctctaaggaggttataaaaa |
| B0029 | tctagagttcacacaggaaacctactag |
| B0032 | tctagagtcacacaggaaagtactag |
| B0035 | tctagagattaaagaggagaatactag |
| RBS36 | ctagtatttctcctgtgtgaactctaga |
| RBS_luxI_ | gaattcattaaagaggagaaaggtacc |
| lldRO | tggtctgacca |
| *aav* | gctgcaaacgacgaaaactacgctgccgcagtt |
| *laa* | gctgcaaacgacgaaaactacgctttagccgca |
| *lldR* | atgagcgttaaagcgcatgaaagcgtgatggattgggttaccgaagaactgcgcagcggtcgcctgaaaattggtgatcatctgccgagcgaacgtgccctgagcgaaacactgggtgtgagccgtagcagcctgcgtgaagcactgcgtgtgctggaagcactgggtactattagcaccgccacgggcagcggtccacgtagtggtacaattattaccgcagcaccgggtcaggcactgagcctgagtgttaccctgcagctggttacgaatcaggttggtcatcatgatatttatgaaacccgtcagctgctggaaggttgggcagcactgcatagcagcgccgaacgtggtgattgggatgttgcggaagcactgctggaaaaaatggatgatccgaccctgccgctggaagattttctgcgttttgatgcggaatttcatgttgtcattagcaaaggtgcagaaaatccgctgattagtacactgatggaagcactgcgcctgagcgttgcagatcataccgttgcacgtgccctggccctgcctgattggcctgcaacaagcgcacgcctgcagaaagaacatcgtgccattctggccgctctgcgtgcaggtgaaagcacaatggcagcaaccctgattaaagatcatatcgaaggttattatcaggaaaccgcagcagcagaagcataa |
| *sfGFP* | atgcgtaaaggcgaagagctgttcactggtgtcgtccctattctggtggaactggatggtgatgtcaacggtcataagttttccgtgcgtggcgagggtgaaggtgacgcaactaatggtaaactgacgctgaagttcatctgtactactggtaaactgccggtaccttggccgactctggtaacgacgctgacttatggtgttcagtgctttgctcgttatccggaccatatgaagcagcatgacttcttcaagtccgccatgccggaaggctatgtgcaggaacgcacgatttcctttaaggatgacggcacgtacaaaacgcgtgcggaagtgaaatttgaaggcgataccctggtaaaccgcattgagctgaaaggcattgactttaaagaagacggcaatatcctgggccataagctggaatacaattttaacagccacaatgtttacatcaccgccgataaacaaaaaaatggcattaaagcgaattttaaaattcgccacaacgtggaggatggcagcgtgcagctggctgatcactaccagcaaaacactccaatcggtgatggtcctgttctgctgccagacaatcactatctgagcacgcaaagcgttctgtctaaagatccgaacgagaaacgcgatcatatggttctgctggagttcgtaaccgcagcgggcatcacgcatggtatggatgaactgtacaaatga |
| *luxA* | atgaaatttggaaactttttgcttacataccaacctccccaattttctcaaacagaggtaatgaaacgtttggttaaattaggtcgcatctctgaggagtgtggttttgataccgtatggttactggagcatcatttcacggagtttggtttgcttggtaacccttatgtcgctgctgcatatttacttggcgcgactaaaaaattgaatgtaggaactgccgctattgttcttcccacagcccatccagtacgccaacttgaagatgtgaatttattggatcaaatgtcaaaaggacgatttcggtttggtatttgccgagggctttacaacaaggactttcgcgtattcggcacagatatgaataacagtcgcgccttagcggaatgctggtacgggctgataaagaatggcatgacagagggatatatggaagctgataatgaacatatcaagttccataaggtaaaagtaaaccccgcggcgtatagcagaggtggcgcaccggtttatgtggtggctgaatcagcttcgacgactgagtgggctgctcaatttggcctaccgatgatattaagttggattataaatactaacgaaaagaaagcacaacttgagctttataatgaagtggctcaagaatatgggcacgatattcataatatcgaccattgcttatcatatataacatctgtagatcatgactcaattaaagcgaaagagatttgccggaaatttctggggcattggtatgattcttatgtgaatgctacgactatttttgatgattcagaccaaacaagaggttatgatttcaataaagggcagtggcgtgactttgtattaaaaggacataaagatactaatcgccgtattgattacagttacgaaatcaatcccgtgggaacgccgcaggaatgtattgacataattcaaaaagacattgatgctacaggaatatcaaatatttgttgtggatttgaagctaatggaacagtagacgaaattattgcttccatgaagctcttccagtctgatgtcatgccatttcttaaagaaaaacaacgttcgctattatattag |
| *luxB* | atgaaatttggattgttcttccttaacttcatcaattcaacaactgttcaagaacaaagtatagttcgcatgcaggaaataacggagtatgttgataagttgaattttgaacagattttagtgtatgaaaatcatttttcagataatggtgttgtcggcgctcctctgactgtttctggttttctgctcggtttaacagagaaaattaaaattggttcattaaatcacatcattacaactcatcatcctgtccgcatagcggaggaagcttgcttattggatcagttaagtgaagggagatttattttagggtttagtgattgcgaaaaaaaagatgaaatgcatttttttaatcgcccggttgaatatcaacagcaactatttgaagagtgttatgaaatcattaacgatgctttaacaacaggctattgtaatccagataacgatttttatagcttccctaaaatatctgtaaatccccatgcttatacgccaggcggacctcggaaatatgtaacagcaaccagtcatcatattgttgagtgggcggccaaaaaaggtattcctctcatctttaagtgggatgattctaatgatgttagatatgaatatgctgaaagatataaagccgttgcggataaatatgacgttgacctatcagagatagaccatcagttaatgatattagttaactataacgaagatagtaataaagctaaacaagagacgcgtgcatttattagtgattatgttcttgaaatgcaccctaatgaaaatttcgaaaataaacttgaagaaataattgcagaaaacgctgtcggaaattatacggagtgtataactgcggctaagttggcaattgaaaagtgtggtgcgaaaagtgtattgctgtcctttgaaccaatgaatgatttgatgagccaaaaaaatgtaatcaatattgttgatgataatattaagaagtaccacatggaatatacctaa |
| *luxC* | atgactaaaaaaatttcattcattattaacggccaggttgaaatctttcccgaaggtgatgatttagtgcaatccattaattttggtgataatagtgtttacctgccaatattgaatgactctcatgtaaaaaacattattgattgtaatggaaataacgaattacggttgcataacattgtcaattttctctatacggtagggcaaagatggaaaaatgaagaatactcaagacgcaggacatacattcgtgacttaaaaaaatatatgggatattcagaagaaatggctaagctagaggccaattggatatctatgattttatgttctaaaggcggcctttatgatgttgtagaaaatgaacttggttctcgccatatcatggatgaatggctacctcaggatgaaagttatgttcgggcttttccgaaaggtaaatctgtacatctgttggcaggtaatgttccattatctgggatcatgtctatattacgcgcaattttaactaagaatcagtgtattataaaaacatcgtcaaccgatccttttaccgctaatgcattagcgttaagttttattgatgtagaccctaatcatccgataacgcgctctttatctgttatatattggccccaccaaggtgatacatcactcgcaaaagaaattatgcaacatgcggatgttattgtcgcttggggagggccagatgcgattaattgggcggtagagcatgcgccatcttatgctgatgtgattaaatttggttctaaaaagagtctttgcattatcgataatcctgttgatttgacgtccgcagcgacaggtgcggctcatgatgtttgtttttacgatcagcgagcttgtttttctgcccaaaacatatattacatgggaaatcattatgaggaatttaagttagcgttgatagaaaaacttaatctatatgcgcatatattaccgaatgccaaaaaagattttgatgaaaaggcggcctattctttagttcaaaaagaaagcttgtttgctggattaaaagtagaggtggatattcatcaacgttggatgattattgagtcaaatgcaggtgtggaatttaatcaaccacttggcagatgtgtgtaccttcatcacgtcgataatattgagcaaatattgccttatgttcaaaaaaataagacgcaaaccatatctatttttccttgggagtcatcatttaaatatcgagatgcgttagcattaaaaggtgcggaaaggattgtagaagcaggaatgaataacatatttcgagttggtggatctcatgacggaatgcgaccgttgcaacgattagtgacatatatttctcatgaaaggccatctaactatacggctaaggatgttgcggttgaaatagaacagactcgattcctggaagaagataagttccttgtatttgtcccataa |
| *luxD* | atggaaaatgaatcaaaatataaaaccatcgaccacgttatttgtgttgaaggaaataaaaaaattcatgtttgggaaacgctgccagaagaaaacagcccaaagagaaagaatgccattattattgcgtctggttttgcccgcaggatggatcattttgctggtctggcggaatatttatcgcggaatggatttcatgtgatccgctatgattcgcttcaccacgttggattgagttcagggacaattgatgaatttacaatgtctataggaaagcagagcttgttagcagtggttgattggttaactacacgaaaaataaataacttcggtatgttggcttcaagcttatctgcgcggatagcttatgcaagcctatctgaaatcaatgcttcgtttttaatcaccgcagtcggtgttgttaacttaagatattctcttgaaagagctttagggtttgattatctcagtctacccattaatgaattgccgaataatctagattttgaaggccataaattgggtgctgaagtctttgcgagagattgtcttgattttggttgggaagatttagcttctacaattaataacatgatgtatcttgatataccgtttattgcttttactgcaaataacgataattgggtcaagcaagatgaagttatcacattgttatcaaatattcgtagtaatcgatgcaagatatattctttgttaggaagttcgcatgacttgagtgaaaatttagtggtcctgcgcaatttttatcaatcggttacgaaagccgctatcgcgatggataatgatcatctggatattgatgttgatattactgaaccgtcatttgaacatttaactattgcgacagtcaatgaacgccgaatgagaattgagattgaaaatcaagcaatttctctgtcttaa |
| *luxE* | atgaagggtataaaagagtatgacagcagtgctgccatactttctaatattatcttgaggagtaaaacaggtatgacttcatatgttgataaacaagaaattacagcaagctcagaaattgatgatttgattttttcgagcgatccattagtgtggtcttacgacgagcaggaaaaaatcagaaagaaacttgtgcttgatgcatttcgtaatcattataaacattgtcgagaatatcgtcactactgtcaggcacacaaagtagatgacaatattacggaaattgatgacatacctgtattcccaacatcggtttttaagtttactcgcttattaacttctcaggaaaacgagattgaaagttggtttaccagtagcggcacgaatggtttaaaaagtcaggtggcgcgtgacagattaagtattgagagactcttaggctctgtgagttatggcatgaaatatgttggtagttggtttgatcatcaaatagaattagtcaatttgggaccagatagatttaatgctcataatatttggtttaaatatgttatgagtttggtggaattgttatatcctacgacatttaccgtaacagaagaacgaatagattttgttaaaacattgaatagtcttgaacgaataaaaaatcaagggaaagatctttgtcttattggttcgccatactttatttatttactctgccattatatgaaagataaaaaaatctcattttctggagataaaagcctttatatcataaccggaggcggctggaaaagttacgaaaaagaatctctgaaacgtgatgatttcaatcatcttttatttgatactttcaatctcagtgatattagtcagatccgagatatatttaatcaagttgaactcaacacttgtttctttgaggatgaaatgcagcgtaaacatgttccgccgtgggtatatgcgcgagcgcttgatcctgaaacgttgaaacctgtacctgatggaacgccggggttgatgagttatatggatgcgtcagcaaccagttatccagcatttattgttaccgatgatgtcgggataattagcagagaatatggtaagtatcccggcgtgctcgttgaaattttacgtcgcgtcaatacgaggacgcagaaagggtgtgctttaagcttaaccgaagcgtttgatagttga |
| *asd* | atgtgccaggaggagaccggcacatttatacagcacacatctttgcaggaaaaaacgcttatgaaaaatgttggttttatcggctggcgcggtatggtcggctccgttctcatgcaacgcatggttgaagagcgcgacttcgacgccattcgccctgtcttcttttctacttctcagcttggtcaggctgcgccgtcttttggcggaaccactggcacacttcaggatgcctttgatctggaggcgctaaaggccctcgatatcattgtgacctgtcagggcggcgattataccaacgaaatctatccaaagcttcgtgaaagcggatggcaaggttactggattgacgcagcatcatctctgcgcatgaaagatgacgccatcatcattcttgaccccgtcaatcaggacgtcattaccgacggattaaataatggcatcaggacttttgttggcggtaactgtaccgtaagcctgatgttgatgtcgctgggtggtttattcgccaatgatcttgttgattgggtgtccgttgcaacctaccaggccgcttccggcggtggtgcgcgacatatgcgtgagttattaacccaaatgggccatctgtatggccatgtggcagatgaactcgcgaacccgtcctctgctattctcgatatcgaacgcaaagtcacaaccttaacccgtagcggtgagctgccggtagataactttggcgtgccgctggcgggtagcctgattccgtggatcgacaaacagcttgataacggtcagagccgcgaagagtggaaagggcaggcggaaaccaacaagatcctcaacacatcttccgtaattccggtagatggtttatgtgtgcgtgtcggggcattgcgctgccacagccaggcattcactattaaattgaaaaaagatgtgtctattccgaccgtggaagaactgctggctgcgcacaatccgtgggcgaaagttgttccaaacgatcgggaaatcactatgcgtgagctaaccccagctgccgttaccggcacgctgaccacgccggtaggccgtctgcgtaagctgaatatgggaccagagttcctgtcagcctttaccgtgggcgaccagctgctgtggggggccgcggagccgctgcgtcggatgcttcgtcaactggcgtaa |
| *coa* | atgaaaaagcagatcatcagcctgggtgcactggcagtagcaagcagcctgtttacctgggataacaaagcagatgcaattgtgaccaaagattatagcggtaaaagccaggttaatgcaggtagcaaaaacggcaccctgattgatagtcgttatctgaatagcgcactgtattatctggaagattatattatctatgcaatcggtctgaccaataaatatgaatatggtgataacatctataaagaagcaaaagatcgtctgctggaaaaagttctgcgtgaagatcagtatctgctggaacgtaaaaagagccagtatgaagattataaacagtggtatgcgaattataaaaaggaaaatccgcgtacagatctgaaaatggccaactttcataaatataacctggaagaactgagcatgaaagaatataatgaactgcaggatgcactgaaacgtgcactggatgattttcatcgcgaagttaaagatattaaagataaaaacagcgatctgaaaacctttaatgcagcagaagaagataaagcaaccaaagaagtatatgatctggtgtctgaaatcgatacactggtcgtaagctattatggtgataaagattatggcgaacatgccaaagaactgcgtgcaaaactggatctgatcctgggcgatacggataatcctcataaaattaccaacgaacgtattaaaaaggaaatgatcgatgatctgaacagcattattgatgatttttttatggaaaccaaacagaaccgtccgaaaagcattacaaaatataacccgaccacccataactataaaaccaacagcgataacaaaccgaattttgataaactggtggaagaaaccaaaaaggcagtcaaagaagcagatgatagctggaaaaagaaaaccgtgaaaaagtatggtgaaaccgaaaccaaaagcccggtagtgaaagaagaaaaaaaagttgaagaaccgcaggcaccgaaagttgataatcagcaggaagttaaaaccaccgcaggtaaagcagaagaaaccacccagccggttgcacagccgctggttaaaattccgcagggtaccattaccggtgaaattgttaaaggtccggaatatccgacaatggaaaataaaaccgttcagggtgaaattgttcagggtccggattttctgacaatggaacagagcggtccgagcctgagcaataattataccaatccgccgctgaccaatccgattctggaaggtctggaaggtagcagcagcaaactggaaattaaaccgcagggtaccgaaagcaccctgaaaggtacccagggtgaaagcagcgatattgaagttaaaccgcaggcaaccgaaaccaccgaagcaagccagtatggtccgcgtgttaccaaataa |
| luxI | atgactataatgataaaaaaatcggattttttggcaattccatcggaggagtataaaggtattctaagtcttcgttatcaagtgtttaagcaaagacttgagtgggacttagttgtagaaaataaccttgaatcagatgagtatgataactcaaatgcagaatatatttatgcttgtgatgatactgaaaatgtaagtggatgctggcgtttattacctacaacaggtgattatatgctgaaaagtgtttttcctgaattgcttggtcaacagagtgctcccaaagatcctaatatagtcgaattaagtcgttttgctgtaggtaaaaatagctcaaagataaataactctgctagtgaaattacaatgaaactatttgaagctatatataaacacgctgttagtcaaggtattacagaatatgtaacagtaacatcaacagcaatagagcgatttttaaagcgtattaaagttccttgtcatcgtattggagacaaagaaattcatgtattaggtgatactaaatcggttgtattgtctatgcctattaatgaacagtttaaaaaagcagtcttaaattaa |
| luxR | atgatatataacacgcaaaacttgcgacaaacaataggtaaggataaagagatgggtatgaaaaacataaatgccgacgacacatacagaataattaataaaattaaagcttgtagaagcaataatgatattaatcaatgcttatctgatatgactaaaatggtacattgtgaatattatttactcgcgatcatttatcctcattctatggttaaatctgatatttcaattctagataattaccctaaaaaatggaggcaatattatgatgacgctaatttaataaaatatgatcctatagtagattattctaactccaatcattcaccaattaattggaatatatttgaaaacaatgctgtaaataaaaaatctccaaatgtaattaaagaagcgaaaacatcaggtcttatcactgggtttagtttccctattcatacggctaacaatggcttcggaatgcttagttttgcacattcagaaaaagacaactatatagatagtttatttttacatgcgtgtatgaacataccattaattgttccttctctagttgataattatcgaaaaataaatatagcaaataataaatcaaacaacgatttaaccaaaagagaaaaagaatgtttagcgtgggcatgcgaaggaaaaagctcttgggatatttcaaaaatattaggctgcagtgagcgtactgtcactttccatttaaccaatgcgcaaatgaaactcaatacaacaaaccgctgccaaagtatttctaaagcaattttaacaggagcaattgattgcccatactttaaaaattaa |
| *SAH* | atggactacaaggacgacgatgacaagaaaacccgtatcgtttcttctgttaccaccaccctgctgctgggctctatcctgatgaacccggttgcgaacgcggcggactctgacatcaacatcaaaaccggcaccaccgacatcggctctaacaccaccgttaaaaccggcgacctggttacctacgacaaagaaaacggcatgcacaaaaaagttttctactctttcatcgacgacaaaaaccacaacaaaaaactgctggttatccgtaccaaaggcaccatcgctggccagtaccgtgtatacagcgaagaaggcgcgaacaaatctggcctggcgtggccgtctgcgttcaaagttcagctgcagctgccggacaacgaagttgcgcagatatctgactactacccgcgtaactctatcgacaccaaagaatacatgtctaccctgacctacggcttcaacggcaacgttaccggcgacgacaccggcaaaatcggcggcctgataggcgcgaacgtaagcatcggccacaccctgaaatacgttcagccggacttcaaaaccatcctggaatctccgaccgacaaaaaagttggctggaaagttatcttcaacaacatggttaaccagaactggggcccgtacgaccgtgactcttggaacccggtttacggcaaccagctgttcatgaaaacccgtaacggctctatgaaagcggcggaaaacttcctggacccgaacaaagcgtcttctctgctgtcttctggcttctctccggacttcgcgaccgttatcactatggaccgtaaagcgtctaaacagcagaccaacatcgacgttatctacgaacgtgttcgtgacgactaccagctgcactggacctctaccaactggaaaggcaccaacaccaaagacaaatggaccgaccgttcttctgaacgttacaaaatcgactgggaaaaagaagaaatgaccaactaa |
